# Label-free detection of polystyrene nanoparticles in *Daphnia magna* using Raman confocal mapping †

**DOI:** 10.1039/d3na00323j

**Published:** 2023-05-30

**Authors:** Jasreen Kaur, Egle Kelpsiene, Govind Gupta, Illia Dobryden, Tommy Cedervall, Bengt Fadeel

**Affiliations:** a Division of Molecular Toxicology, Institute of Environmental Medicine, Karolinska Institutet Nobels väg 13 171 77 Stockholm Sweden bengt.fadeel@ki.se; b NanoLund, Department of Biochemistry and Structural Biology, Lund University Lund Sweden; c Department of Material and Surface Design, RISE Research Institutes of Sweden Stockholm Sweden

## Abstract

Micro- and nanoplastic pollution has emerged as a global environmental problem. Moreover, plastic particles are of increasing concern for human health. However, the detection of so-called nanoplastics in relevant biological compartments remains a challenge. Here we show that Raman confocal spectroscopy-microscopy can be deployed for the non-invasive detection of amine-functionalized and carboxy-functionalized polystyrene (PS) nanoparticles (NPs) in *Daphnia magna*. The presence of PS NPs in the gastrointestinal (GI) tract of *D. magna* was confirmed by using transmission electron microscopy. Furthermore, we investigated the ability of NH_2_-PS NPs and COOH-PS NPs to disrupt the epithelial barrier of the GI tract using the human colon adenocarcinoma cell line HT-29. To this end, the cells were differentiated for 21 days and then exposed to PS NPs followed by cytotoxicity assessment and transepithelial electrical resistance measurements. A minor disruption of barrier integrity was noted for COOH-PS NPs, but not for the NH_2_-PS NPs, while no overt cytotoxicity was observed for both NPs. This study provides evidence of the feasibility of applying label-free approaches, *i.e.*, confocal Raman mapping, to study PS NPs in a biological system.

## Abstract 2

Polystyrene nanoparticles can be detected in crustaceans in a label-free manner by confocal Raman mapping and this was further confirmed by TEM analysis.

## Graphical abstract

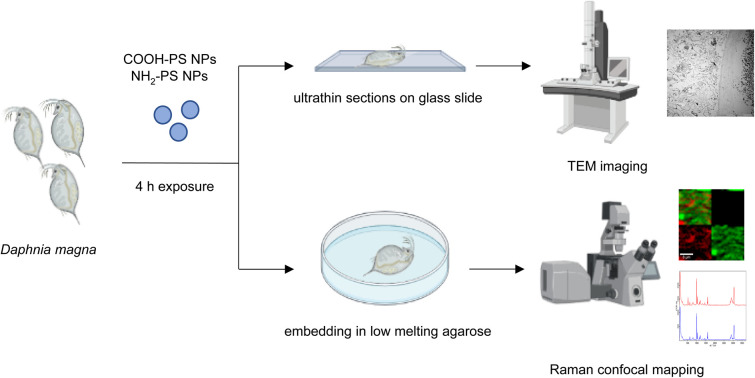

## 1. Introduction

The production of plastic since the 1950s has surpassed that of almost every other man-made material,^1^ and enormous amounts of plastic fragments are accumulating in the marine environment and in other habitats.^2,3^ Moreover, the weathering of such fragments may lead to a substantial environmental burden of nanoscale pieces of plastics (aka nanoplastics).^4^ The definition of nanoplastics is still under debate, and it has been pointed out recently that incidentally produced nanoplastics are heterogeneous with respect to composition and morphology unlike most engineered nanomaterials.^5^ Notwithstanding, synthetic (usually spherical) particles of polystyrene (PS) are frequently used as a proxy for micro- and nanoplastic environmental debris.^6^ Notably, we have shown that the mechanical breakdown of PS-based single-use plastics (SUPs) for just 5 min gives rise to nanoparticles with remarkably narrow size distributions including spherical particles with a mean diameter of approximately 125 nm (by TEM).^7^ This thus lends support to the use of PS NPs as “surrogate” materials.^8^

Numerous studies have addressed the potential environmental impact of PS NPs using model organisms such as crustaceans and fish.^9–11^ Moreover, evidence of trophic transfer (transfer along the food chain) has been provided.^12,13^ Human exposure also seems plausible, and a recent study implied, on the basis of micro-Fourier transform infrared (μFTIR) analysis, that microplastics can be found in human lung tissue samples.^14^ Using mass spectrometry-based protocols to screen human blood samples, other investigators suggested that plastic “pollution” is also present in the blood, perhaps due to a translocation of particles following ingestion or inhalation, but great care is needed to exclude sample contamination.^15,16^ In fact, these studies serve to highlight one of the major challenges in the field, that is, the reliable detection of micro- and nanoplastics in biological matrices. Hence, “adequate analytical tools to sample, isolate, detect, quantify, and characterize small microplastics, especially nano-sized plastic particles, are urgently needed”.^17^ Raman spectroscopy, which provides information on the structural “fingerprint” of molecules, has been successfully applied for the detection of microplastics (in water).^18^ Moreover, a recent study demonstrated the feasibility of identifying aggregates of nanosized (100 ± 10 nm) PS particles using Raman spectroscopy.^19^ However, the label-free detection (and localization) of nanoplastics *in situ* (that is, in tissues) remains a major challenge. Here we performed confocal Raman microscopy to identify NH_2_-PS and COOH-PS NPs (200 nm) in the freshwater crustacean *Daphnia magna*. To this end, we used a well-established model of exposure in which the daphnids were allowed to filter the water containing PS NPs (or water alone).^20^ Transmission electron microscopy was performed to verify the presence of the NPs in these organisms. We also evaluated the possible effects of the NPs on barrier integrity using a human *in vitro* model of the gastrointestinal epithelium.

## 2. Experimental section

### 2.1. NP characterization

Aminated PS NPs, PS-NH_2_ NPs (200 nm according to the manufacturer) (PA02001-PA04001), and carboxylated PS NPs, PS-COOH NPs (200 nm according to the manufacturer) (PC02001-PC07003) were purchased from Bangs Laboratories, Inc. (Fishers, IN). Prior to the experiments, the NPs were diluted to a concentration of 10 mg mL^−1^ and dialysed against Milli-Q® water for 72 h at 4 °C. The water was changed after 4 h the first day and once a day on the other days.^21^ NPs were characterized using transmission electron microscopy (TEM) and scanning electron microscopy (SEM) to confirm their size and morphology after dialysis. Briefly, 3 μL of each sample was applied on glow-discharged, carbon-coated, and formvar-stabilized 400 mesh copper grids (Ted Pella, Inc., Redding, CA) and incubated for approximately 30 s. Then, the grid was washed once with Milli-Q® water. TEM imaging was performed using the Hitachi HT7700 (Hitachi High-Technologies) microscope at 100 kV operating with a 2kx2k Veleta CCD camera (Olympus Soft Imaging System). For SEM, samples were applied onto 0.1 mm polyethersulfone polymer (PES) filters and air-dried. The membranes were mounted on specimen stubs using carbon adhesive tabs and sputter coated with platinum (Quorum Q150T ES). SEM images were acquired using an Ultra 55 field emission scanning electron microscope (Zeiss, Oberkochen, Germany) at 5 kV using the InLens detector. Furthermore, the *ζ*-potential in Milli-Q® water (as a reference), tap water (used for *D. magna* experiments), and RPMI-1640 medium with and without 10% FBS (used for experiments with HT-29 cells) was determined for both NPs using a Zetasizer Nano ZS instrument (Malvern Instruments, Worcestershire, UK).

### 2.2. *D. magna* studies

*D. magna* originating from Lake Bysjön (55°40′31.3′′N 13°32′41.9′′E) kept under controlled laboratory conditions for several hundred generations were used in the present study. For all experiments, *D. magna* were maintained at 18 °C under an 8 : 16 h light/dark photoperiod. *D. magna* adults were kept in clean tap water for 24 h prior to incubation with NPs to allow evacuation of remaining algal cells from the gut. During the exposure, *D. magna* (*n* = 15 individuals per tube) were placed into 15 mL tubes (in total there were four replicates for each group) containing a total volume of 5 mL of tap water and 0 mg L^−1^ (control group) and 224 mg L^−1^ of PS nanoparticles, as described.^20^ The daphnids were allowed to filter the water containing PS-NH_2_ NPs, PS-COOH NPs or water alone for 4 h. The time-point was chosen to avoid toxicity/mortality of the NPs.^20^ The selected dose is the highest non-lethal dose that could be achieved (at 4 h).

### 2.3. Human cell studies

The human colorectal adenocarcinoma cell line HT-29 (ATCC HTB-38) was grown in RPMI-1640 medium supplemented with 10% fetal bovine serum (FBS), 100 IU mL^−1^ penicillin, 100 μg mL^−1^ streptomycin, and 2 mM l-glutamine in a humidified 5% CO_2_ incubator at 37 °C. Periodical subculturing of the cells was done on average every 4 days. For differentiation, the cells were grown on transwells (Corning™ Falcon™ Cell Culture Inserts) at a density of 1 × 10^5^ cells per mL in 24-well plates (area of the transwell = 0.3 cm^2^) and differentiated with the abovementioned media supplemented with high glucose (10% v/v) for 21 days.^22^ TEER measurements (see below) were done to observe the barrier integrity. The media was replaced every two days. In our pilot studies, cytotoxicity towards HT-29 cells was determined based on LDH release using the CytoTox 96® Non-Radioactive Cytotoxicity Assay (Promega). To this end, cells were seeded in a 96-well plate at a density of 5 × 10^5^ cells per mL without differentiation (no glucose) for 24 h and then exposed to a range of concentrations (0.1–100 μg mL^−1^) of COOH-PS NPs and NH_2_-PS NPs. After exposure, supernatants were collected and processed for LDH release according to the manufacturer's instructions. The samples were analyzed on a Tecan Infinite® F200 spectrophotometer (Männedorf, Switzerland).

### 2.4. TEM analysis

TEM imaging was performed on *D. magna* samples after exposure to COOH-PS NPs or NH_2_-PS NPs. The samples were prefixed with 4% glutaraldehyde in 0.1 M sodium phosphate buffer pH 7.4 for 1 h at 4 °C. After that they were fixed in 1% OsO_4_ in 0.1 sodium phosphate buffer for 1 h at 4 °C and were subsequently dehydrated using a gradient of ethanol followed by acetone and LX-112 infiltration followed by embedding in LX-112 resin (Ladd Research Industries, Vermont, OH). Ultrathin sections (50–80 nm) were prepared using a Leica EM UC6 microtome and these were further contrasted with uranyl acetate followed by lead citrate, and finally examined using a Hitachi HT 7700 electron microscope (Hitachi High-Technologies). We used the 2kx2k Veleta CCD camera (Olympus) for image acquisition. TEM analysis was also performed on HT-29 cells. Briefly, cells were detached from the transwells after cell culture and prefixed with 4% glutaraldehyde in 0.1 M sodium phosphate buffer pH 7.4 for 1 h at 4 °C. Thereafter, the procedure described for *D. magna* samples was followed.

### 2.5. Raman mapping

Raman spectroscopy and confocal mapping was carried out using a WITec Alpha 300 RAS system (Oxford Instruments, Ulm, Germany) equipped with a 532 nm excitation laser. The measurements were performed using a 50x ZEISS LD EC Epiplan-Neofluar Dic 50x/NA 0.55 objective. Laser power was optimized to minimize heat induced effects and set at 10 mW. The spectrometer diffraction grating of 600 L mm^−1^ was implemented. First, *D. magna* exposed to the COOH-PS NPs or NH_2_-PS NPs as indicated were embedded in low-melting agarose^23^ (Sigma, Sweden). The gut region of the daphnids was identified and micrographs were captured using an optical microscope (SMZ 1270, Nikon). Next, the samples were used for the confocal Raman analysis. The Raman spectrum of COOH-PS NPs and NH_2_-PS NPs alone was also recorded as a reference. For the Raman mapping, a Raman spectrum is recorded in every image pixel. In the confocal regime, the focal plane was varied to obtain the signal from the areas of interest inside the daphnids. Then, Raman maps indicating PS intensity distributions were reconstructed based on the evaluated characteristic spectra for all measured pixels. All spectra were analyzed using WITec Project Plus 5.1 software (Oxford Instruments) using cosmic ray removal and baseline correction filters.

### 2.6. TEER analysis

Transepithelial electrical resistance (TEER) was applied for the analysis of epithelial barrier integrity. TEER offers several advantages over other traditional permeability measurements in that it is a rapid, label-free, and noninvasive approach.^24^ HT-29 cells were exposed to COOH-PS NPs or NH_2_-PS NPs as indicated and their barrier integrity was measured using an EVOM^2^ epithelial volt/ohmmeter (World Precision Instruments, Sarasota, FL) operating with STX2 “chopstick” electrodes. The measurements were taken at 0, 2, 4 and 24 h. EGTA (2.5 mM) was used as a positive control. The experiments were performed in biological and technical triplicates. The final TEER values for each condition were calculated using the following formula: TEER value (ohm cm^2^) = (TEER value of sample − TEER value of control) × (area of the transwell).

### 2.7. Cell viability

After exposure to NH_2_-PS and COOH-PS NPs, cytotoxicity was assessed using the Alamar blue assay (Thermo Fisher Scientific, Sweden), as described.^25^ Briefly, the cells grown in transwells were first exposed to the Alamar blue dye directly and incubated for 2 h. The media containing the dye was then removed and added to the 96-well plate. The samples were analyzed on a Tecan Infinite® F200 spectrophotometer (Männedorf, Switzerland). Cell viability was quantified by the % cell viability *versus* the negative control value, which was set as 100%. Lysed cells served as positive control.

### 2.8. Statistical analysis

Data were analyzed using one-way or two-way ANOVA followed by Dunnett's and Tukey's *post hoc* analysis. The *p*-values of *p < 0.05, **p < 0.01, ***p < 0.001, and ****p < 0.0001 were considered significant. The statistical analysis was performed using GraphPad Prism software version 9.0.0 for Windows (GraphPad Software, San Diego, CA).

## 3. Results and discussion

### 3.1. Characterization of PS NPs in relevant test media

PS NPs obtained from a commercial source were characterized with respect to hydrodynamic diameter and *ζ*-potential in water (for *D. magna*) and RPMI-1640 medium with and without serum (for HT-29 cells) (Table S1†). The aminated NPs agglomerated in Milli-Q® water and tap water while this was not the case for the carboxylated NPs. However, both particles showed similar hydrodynamic sizes (270 ± 3 nm for COOH-PS and 213 ± 23 nm for NH_2_-PS NPs) and *ζ*-potential values (−13 ± 1.23 mV and −12.6 ± 0.7 mV for COOH-PS and NH_2_-PS NPs) in RPMI-1640 cell culture medium supplemented with FBS (used for the HT-29 cell culture experiments) (Table S1†). The results for the NH_2_-PS NPs, thought to be positively charged, were unexpected, though it is worth noting that the *ζ*-potential that is exhibited by NPs in suspension does not necessarily align with the surface charge density.^26^ Notwithstanding, this finding is in accordance with our previous work, and may be explained by the lower number of amine groups and the presence of sulfone groups on the surface of the NH_2_-PS NPs,^20^ which may also explain the inferior stability of the NP dispersions in water. However, the presence of serum proteins stabilized the PS NPs. The particles were further characterized using TEM and SEM. The average particle diameter of COOH-PS and NH_2_-PS NPs was 170 nm and 130 nm, respectively (Fig. S1†). SEM confirmed the uniform, spherical morphology of both NPs (Fig. S2†).

### 3.2. Confocal Raman mapping of PS NPs in *D. magna*

**Fig. 1:**
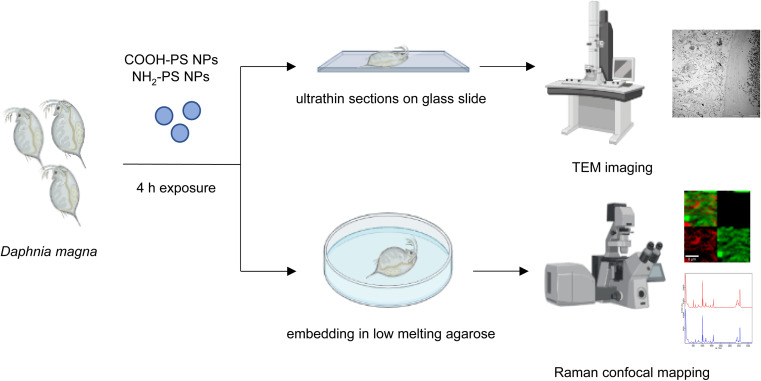
Schematic representation of the experiments performed with *D. magna* and PS NPs.

**Fig. 2:**
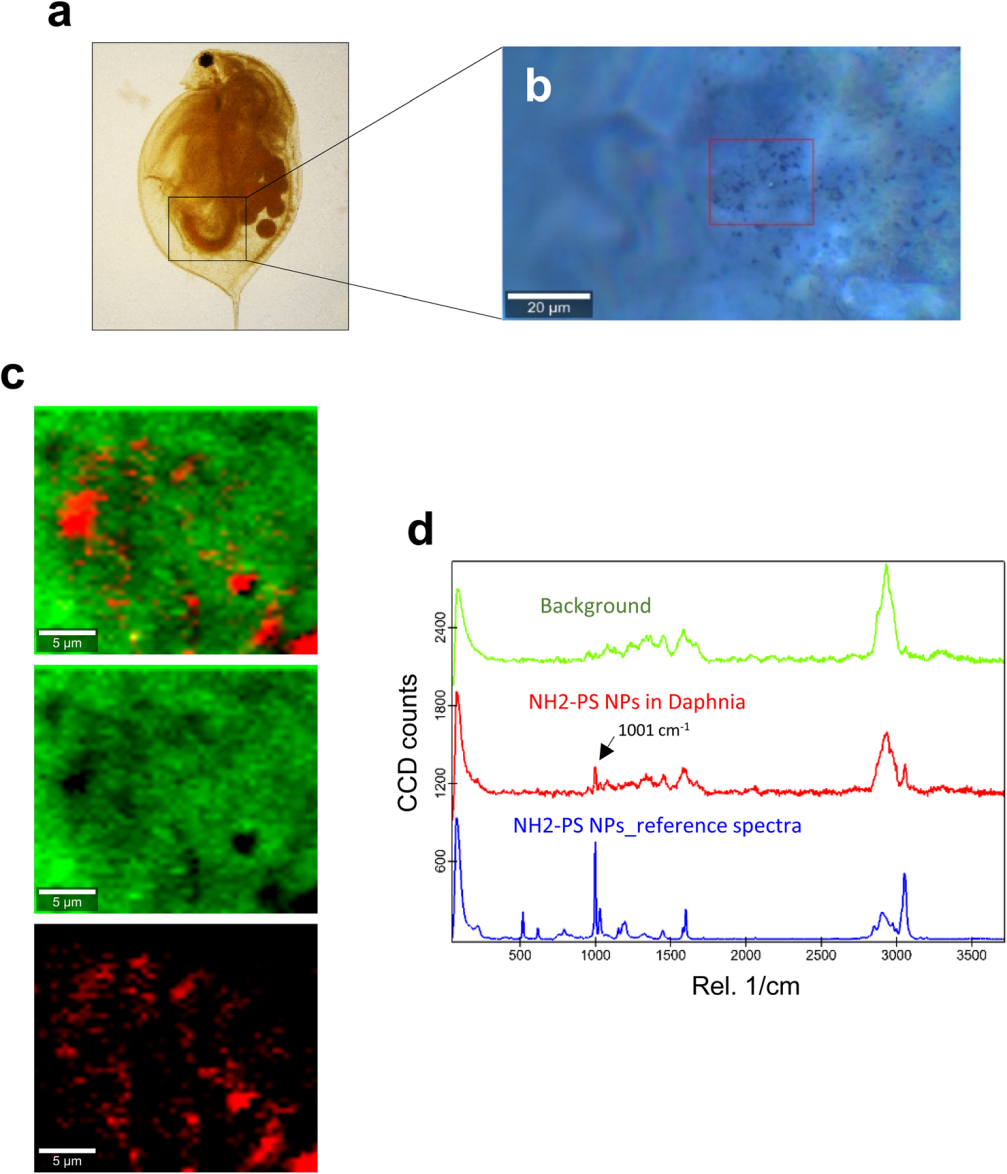
Confocal Raman imaging of NH_2_-PS in the gut of daphnids. (a) Brightfield micrograph of whole daphnia indicating the gut region used for the Raman analysis. (b) An optical image of the scanned area is shown, where the red square shows the exact map position. (c) Reconstructed Raman maps showing the combined distribution map (top) for the identified components NH_2_-PS NPs (red) and the carapace of the daphnid (green). Scale bars: 5 μm. (d) Typical average spectra for NH_2_-PS (red) and background (green) used to create the maps are shown. The 1001 cm^−1^ band specific for PS is identified with an arrow. The blue spectrum is for the reference NH_2_-PS NPs.

**Fig. 3:**
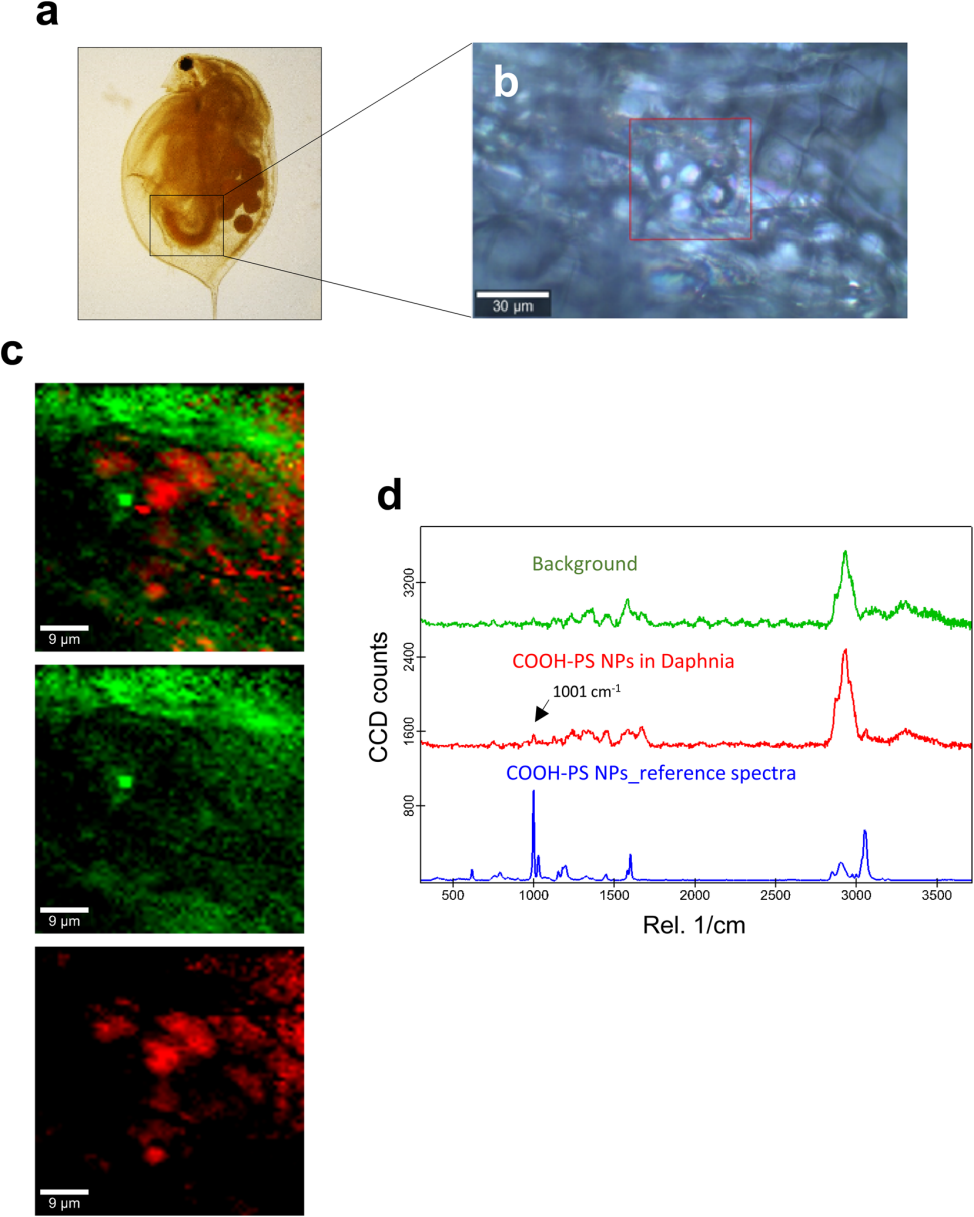
Confocal Raman imaging of COOH-PS in the gut of daphnids. (a) Brightfield micrograph of whole daphnia indicating the gut region used for the Raman analysis. (b) An optical image of the scanned area is shown, where the red square shows the exact map position. (c) Reconstructed Raman maps showing the combined distribution map (top) for the identified components COOH-PS NPs (red) and the carapace of the daphnid (green). Scale bars: 9 μm. (d) Typical average spectra for COOH-PS (red) and background (green) used to construct the maps are shown. The PS-specific 1001 cm^−1^ band is identified with an arrow. The reference spectrum for COOH-PS NPs is shown in blue.

Next, we studied the PS NPs using *D. magna* as a model (Fig. 1). Specifically, we asked whether confocal Raman mapping could be applied to detect the NPs *in situ*, and TEM was applied to verify the findings (see the following section). Previous work has shown that PS NPs exhibit significant functional group-dependent toxicity towards *D. magna* following a 48 h exposure.^27^ However, in the present study, we opted for a brief (4 h) exposure to PS NPs at a concentration (224 mg L^−1^) that would not trigger any toxicity.^20^ First, reference spectra of NH_2_-PS and COOH-PS were obtained (Fig. S3a†). Both spectra displayed a similar chemical fingerprint with almost identical chemical band positions. The main difference is the shoulder peak at around 2938 cm^−1^ possibly due to NH_2_ affecting the CH vibration strength. The characteristic peaks at 1001 cm^−1^ for the aromatic C=C ring breathing mode and 3053 cm^−1^ for CH stretching, previously identified as being PS-specific (in particular, the so-called “identifier peak” at 1001 cm^−1^),^19^ were present in both particles (Fig. S3a†). We also captured the background Raman spectra of unexposed daphnids and could confirm the absence of both peaks at 1001 cm^−1^ and 3053 cm^−1^ in these control samples (Fig. S3b†). Thus, the appearance of such peaks can be attributed to the presence of PS in the sample. Next, confocal Raman mapping was carried out on daphnids exposed to the aminated (NH_2_) PS NPs (Fig. 2a–d). Two components were identified, and their distribution was visualized, *i.e.*, the carapace of the daphnid (shown in green) and the PS component (shown in red). The combined image of both components is shown in Fig. 2c. The corresponding Raman spectra confirmed the presence of PS and both specific peaks at 1001 cm^−1^ and 3053 cm^−1^ were observed for NH_2_-PS NPs (Fig. 2d). Corresponding results were obtained for daphnids exposed to carboxylated (COOH) PS NPs (Fig. 3a–d). Hence, two components were clearly identified, and their distribution was visualized, *i.e.*, the carapace of the daphnid (shown in green) and the PS component (shown in red). The combined image of both components is shown in Fig. 3c, while the corresponding Raman spectra are displayed in Fig. 3d. These results confirmed the presence of PS as both specific peaks at 1001 cm^−1^ and 3053 cm^−1^ were identified for the PS component. Taken together, we have shown that daphnids uptake (ingest) PS nanoparticles with amine and carboxyl modifications. Moreover, it was possible not only to identify the PS NPs (nominally 200 nm), in accordance with a recent study,^19^ but also to demonstrate the presence of these NPs in the digestive tract of daphnids, by exploiting the confocal Raman mapping capability. Such mapping of PS NPs has not been demonstrated before. This label-free approach may yield useful insights regarding the biological impact of PS NPs and other plastic NPs.

### 3.3. TEM to confirm the presence of NPs in *D. magna*

**Fig. 4:**
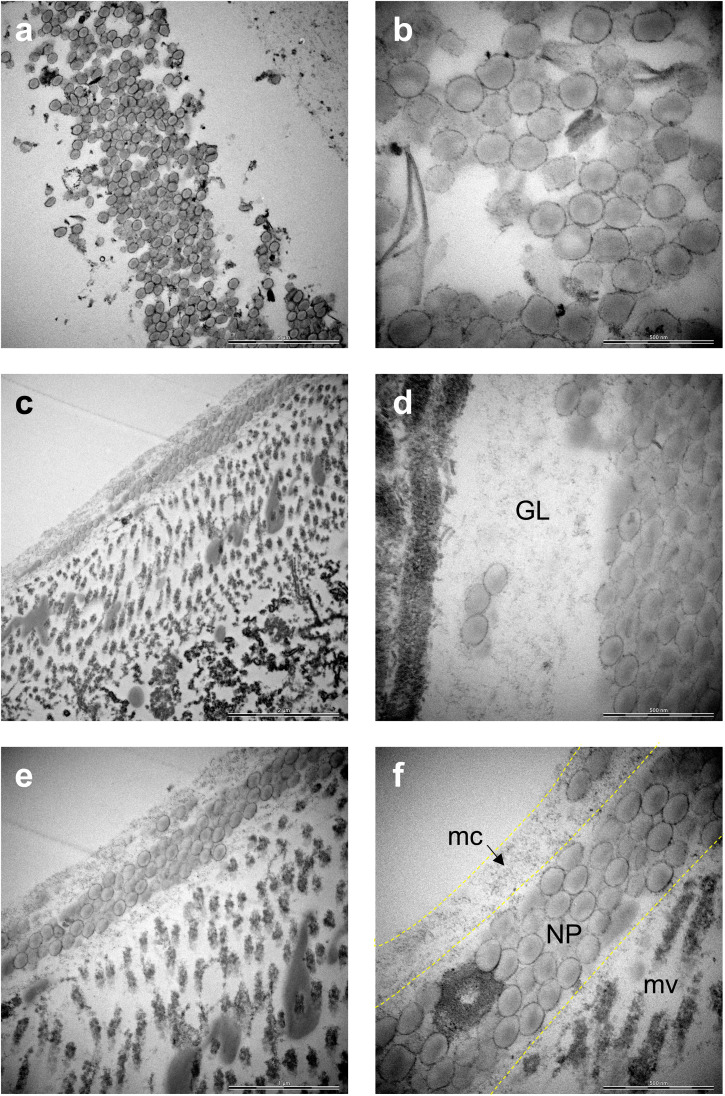
TEM images of the GI tract of daphnids indicating the presence of PS NPs. (a) COOH-PS NPs. Scale bar: 2 μm. (b) COOH-PS NPs. Scale bar: 500 nm. (c) NH_2_-PS NPs. Scale bar: 2 μm. (d) NH_2_-PS NPs. GL, gut lumen. Scale bar: 500 nm. (e) NH_2_-PS NPs. Scale bar: 1 μm. (f) NH_2_-PS NPs. mc, mucus; mv: microvilli. Scale bar: 500 nm.

Raman mapping in combination with bright field imaging suggested the presence of PS NPs in the GI tract. To verify and visualize the presence of particles in the gut, TEM imaging was performed. Both COOH-PS NPs and NH_2_-PS NPs were clearly identified in the exposed individuals. Notably, COOH-PS NPs were only found in the gut lumen (Fig. 4a and b) while the NH_2_-PS NPs were found embedded in both the luminal mucus layer (Fig. 4c) and in the gut lumen (Fig. 4d). Further examples are shown in Fig. 4e and f, clearly demonstrating the presence of NH_2_-PS NPs embedded in the mucus layer residing on top of the microvilli (shown in cross-section) (refer to high-magnification view in Fig. 4f). We could not find any evidence of cellular internalization of NPs or NPs crossing the epithelial barrier; however, we focused only on the GI tract itself and not on other compartments. Other recent work has suggested that PS NPs (50 nm) might cross the intestinal barrier following oral ingestion in mice.^28^ Thus, NPs were found in the mesenteric lymph nodes, liver, and spleen. However, in the latter study, animals were exposed to a so-called “physiological” dose of 2 mg per day (equivalent to the supposed human intake of 5 g of plastic every week) for 6 months. In the present study, daphnids were exposed acutely (4 h) to a relatively low amount of NPs, as the purpose was not to study toxicity, but to test the feasibility of applying a label-free approach for the detection and imaging of NPs *in situ* (in a living organism).

The main component of the gut mucosa is the glycoprotein mucin (containing negatively charged polysaccharide groups) which forms a mesh-like structure of crosslinked and entangled mucin fibers.^29^ Previous work has shown that the type of functional groups present on the surface of NPs plays an important role for their interaction with mucin.^30^ The mechanism involves the electrostatic attraction between positively charged NPs and the polyanionic sites on mucin promoting its aggregation. On the other hand, while negatively charged NPs do not seem to provoke mucin aggregation, they could still adhere to the mucus network, thus affecting its viscosity.^30^ Overall, there is much to learn with respect to the corona formation which may occur in the environment (referred to as the eco-corona) and in different anatomical compartments in the body (bio-corona) (refer to ref. 31 for an excellent overview of this topic). Using *in vitro* and *in vivo* models, Yang *et al.*^32^ provided evidence that the mucin bio-corona may affect translocation of Au NPs across the intestinal epithelium. Moreover, studies have shown that digestive tract fluids may alter the properties of NPs.^33^ Clearly, more work is warranted to understand the fate and behavior of NPs in the gut.

### 3.4. Human *in vitro* model of epithelial barrier integrity

**Fig. 5:**
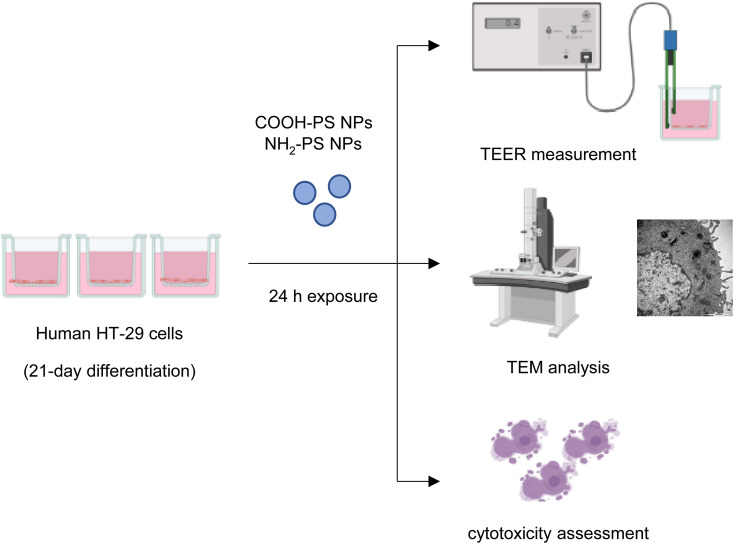
Schematic view of experiments performed using differentiated human HT-29 cells.

Microplastics are suspected to cause intestinal barrier dysfunction and/or changes in the intestinal microenvironment (reviewed in ref. 34). To further study the impact of PS NPs, we set up a human *in vitro* model of the differentiated gastrointestinal epithelium and exposed these epithelial cell layers for 24 h to COOH-PS NPs and NH_2_-PS NPs (Fig. 5). Both NPs (100 μg mL^−1^) were shown to be non-cytotoxic towards these cells (Fig. S4†).

**Fig. 6:**
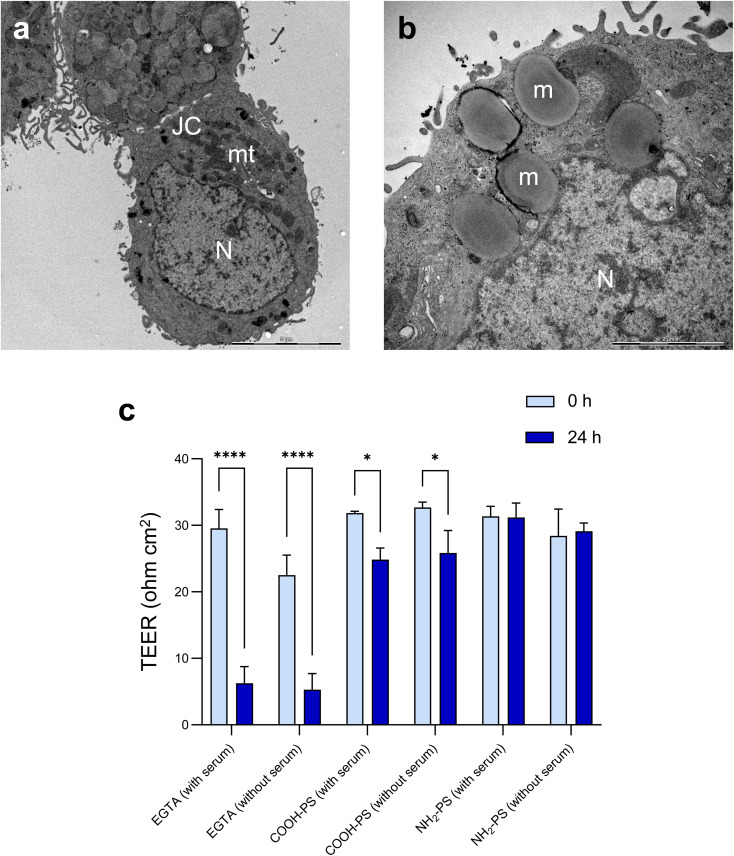
Evaluating barrier integrity following exposure to PS NPs. (a and b) TEM micrographs of differentiated human HT-29 cells. (a) JC, junctional complexes (between cells); mt, mitochondria; N, nucleus. Scale bar: 5 μm. (b) m, mucus-filled dense vesicles; N, nucleus. Scale bar: 2 μm. (c) TEER values obtained after exposure of differentiated HT-29 cells to 100 μg mL^−1^ of COOH-PS NPs and NH_2_-PS NPs (in the presence or absence of serum). EGTA (2.5 mM) was used as a positive control. Three independent experiments were performed, and results are shown as mean values ± S.D. Significance was calculated using 2-way ANOVA followed by Tukey's *post hoc* test.

The barrier function of the epithelium is due to the presence of tight junctions between cells. Cell differentiation is further characterized by a brush border (*i.e.*, a microvilli-covered surface) and mucus production, thereby imitating the human gastrointestinal epithelium. We performed TEM to verify ultrastructural features of cell differentiation (Fig. 6a). Then, TEER measurements were performed to assess whether the barrier function was affected by the NPs (in the absence of overt cytotoxicity/cell death). The calcium chelating agent EGTA was used as a positive control. As shown in Fig. 6b, EGTA strongly affected the barrier integrity, as evidenced by a profound decrease in TEER values at 24 h when compared to baseline (0 h). It is noted that each condition (each transwell) serves as its own control as the baseline values may differ between transwells; the results shown are based on three independent experiments. The carboxylated (COOH) PS NPs induced a modest decrease in TEER values while no changes were seen in the presence of the aminated (NH_2_) PS NPs (Fig. 6b). Most NPs promptly bind serum proteins forming a protein bio-corona.^35^ Indeed, the *ζ*-potential of the COOH-PS NPs and NH_2_-PS NPs was equalized in cell culture medium supplemented with FBS indicative of the binding of serum proteins (Table S1†). We also performed TEER measurements following exposure of the monolayers of cells in the absence of FBS. However, similar results were obtained for both NPs (Fig. 6b), implying that the presence (or absence) of serum does not play a significant role in this model.

Previous work has shown that the presence of mucus may affect NP translocation across intestinal epithelial cell barriers.^36^ Specifically, particle size as well as the surface charge may play a role.^37^ We did not investigate the role of mucus specifically, but it is possible that the mucus layer serves to protect the cells from the toxicity of PS NPs. Other investigators have provided evidence of abundant cellular uptake of PS NPs.^38^ However, the latter study was performed using undifferentiated Caco-2 cells which is a convenient model but lacks realism since no mucus is produced by these cells.

## 4. Conclusions

We focused here on confocal Raman imaging of PS NPs in *D. magna*, and on investigating the impact of these NPs on epithelial barrier integrity using a human *in vitro* model. Our study has shown that confocal Raman imaging (mapping) can be exploited as a label-free method to detect PS NPs in intact biological specimens, and TEM was performed to verify the presence of NPs in the gut of exposed daphnids. We observed no or limited toxicity of NH_2_-PS NPs and COOH-PS NPs in our *in vitro* model.

## Author contributions

J. K. performed research, analyzed data, and drafted the manuscript; E. K., G. G. and I. D. performed research and analyzed data; T. C. supervised experiments, and analyzed data; B. F. coordinated the study, supervised the experiments, analyzed data, and edited the manuscript, and all co-authors approved the final version of the paper.

## Conflicts of interest

The authors declare that they have no competing financial interests.

## Supplementary Material

NA-005-D3NA00323J-s001
