# Supplementary material for: Label-free detection of polystyrene nanoparticles in Daphnia magna using Raman confocal mapping
Source: Nanoscale Adv. 2023 May 30;5(13):3453–62. doi: 10.1039/d3na00323j (PMC10295233; doi:10.1039/d3na00323j)
Supplement: NA-005-D3NA00323J-s001 [file NA-005-D3NA00323J-s001.pdf]

Electronic Supplementary Material (ESI) for Nanoscale Advances.
This journal is © The Royal Society of Chemistry 2023

Supplementary information

# Label-free detection of polystyrene nanoparticles in *Daphnia magna* using Raman confocal mapping

J. Kaur[1], E. Kelpsiene[2], G. Gupta[1], I. Dobryden[3], T. Cedervall[2], and B. Fadeel[1]

[1]Division of Molecular Toxicology, Institute of Environmental Medicine, Karolinska Institutet, Stockholm, Sweden.

[2]NanoLund, and Department of Biochemistry and Structural Biology, Lund University, Lund, Sweden.

[3]Department of Material and Surface Design, RISE Research Institutes of Sweden, Stockholm, Sweden.

Table S1. Zeta potential and hydrodynamic size in various media at 100 µg/mL.

| Samples | Zeta potential (mV) | Size (nm) |
|---|---|---|
| COOH PS in Milli-Q® | -37,4 ± 0,72 | 211 ± 1 |
| COOH PS in tap $H_2O$ | -35,7 ± 2,55 | 187 ± 2 |
| COOH PS in RPMI | -17,1 ± 0,6 | 428 ± 31 |
| COOH PS in RPMI + FBS | -13,0 ± 1,23 | 270 ± 3 |
| $NH_2$ PS in Milli-Q® | -18,5 ± 1,2 | 1164 ± 30[a] |
| $NH_2$ PS in tap $H_2O$ | -25,7 ± 0,6 | 1099 ± 265[a] |
| $NH_2$ PS in RPMI | -11,6 ± 0,8 | 811 ± 45 |
| $NH_2$ PS in RPMI + FBS | -12,6 ± 0,7 | 213 ± 23 |

Measurements were done for triplicate samples and data are shown as mean values ± S.D.

[a]Two intensity peaks were identified in these samples; the average values are reported here.

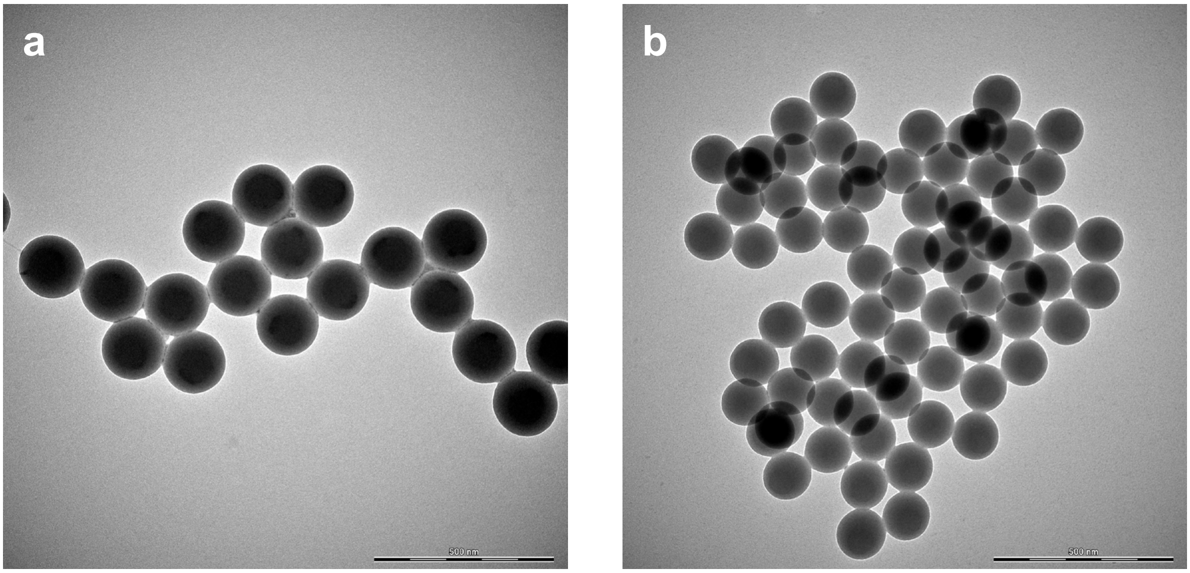


Figure S1. TEM micrographs of (a) PS-COOH NPs (diameter: ~170 nm), and (b) PS-$NH_2$ NPs (diameter: ~130 nm) dispersed in tap water. The particles were adhered onto grids and washed once with Milli-Q® water (no counterstaining). Some particle necking is seen. Scale bars: 500 nm.

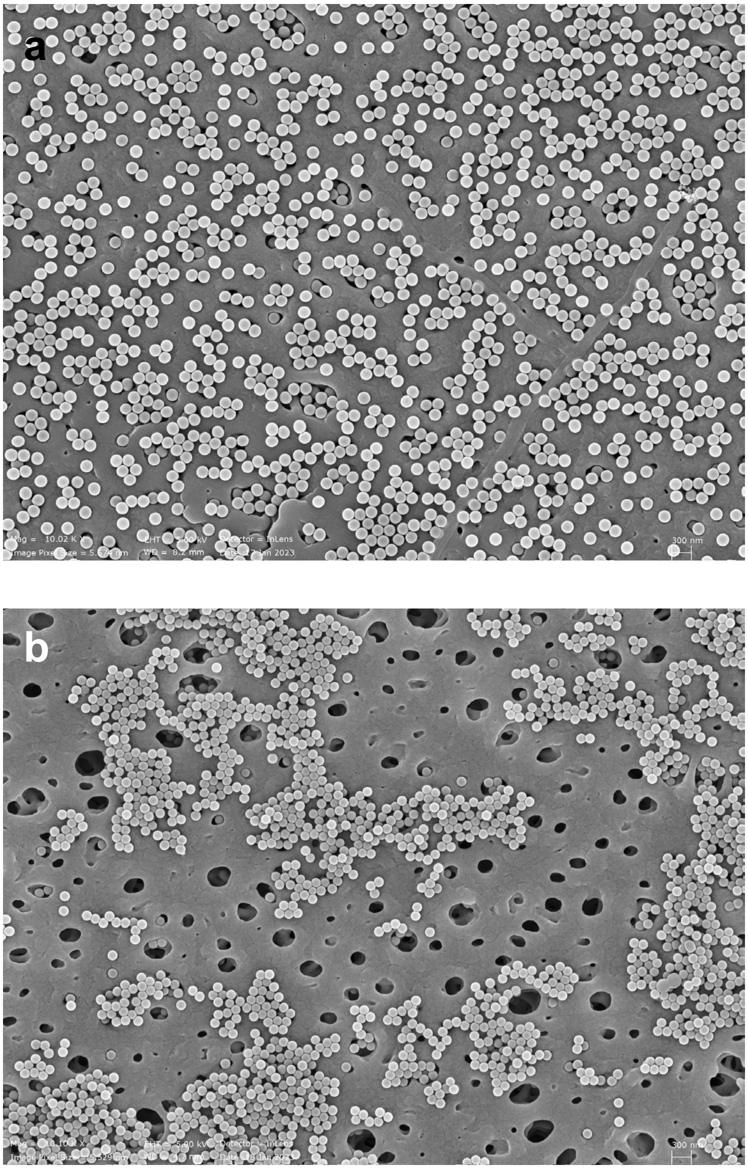


Figure S2. SEM micrographs of (a) PS-COOH NPs, and (b) PS-$NH_2$ NPs. The specimens were sputter coated with platinum prior to imaging as described in the experimental section. Scale bars: 300 nm.

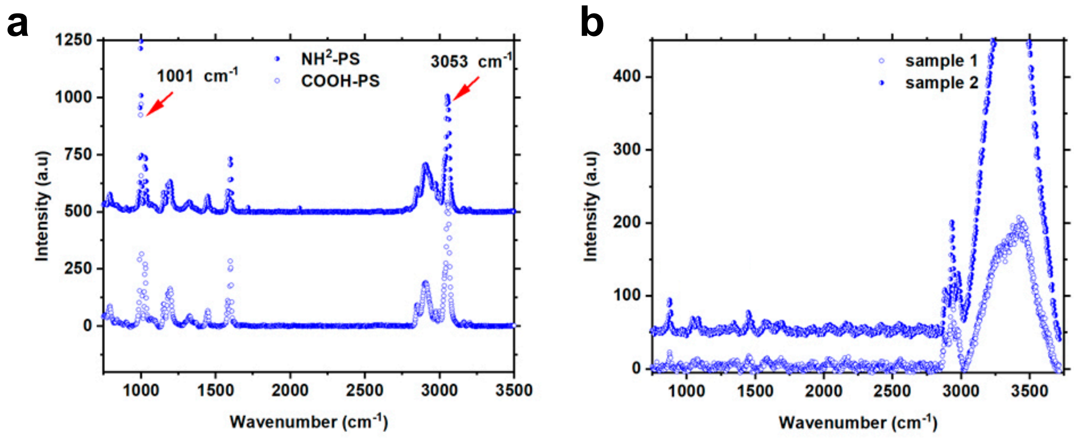


Figure S3. Raman reference spectra measured for PS NPs with COOH (empty circles) and $NH_2$ (half-filled circles) functional groups are shown in panel (a). Both specific peaks for PS at 1001 $cm^{-1}$ and 3053 $cm^{-1}$ are indicated with a red arrow. Spectra measured for control daphnia samples 1 (empty circles) and 2 (half-filled circles) are shown in panel (b). There is no PS specific peak at 1001 $cm^{-1}$ in these controls.

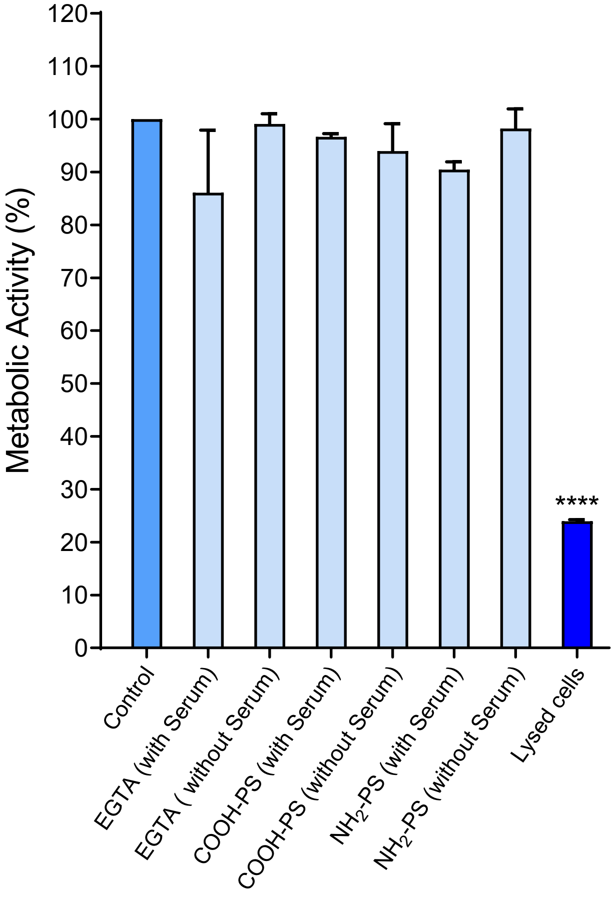


Figure S4. No PS NP-induced cytotoxicity in differentiated HT-29 cells. Human HT-29 cells differentiated for 21 days were exposed for 24 h to 100 mg/mL COOH-PS NPs or $NH_2$-PS NPs in the presence or absence of serum (10% FBS). Cell viability (metabolic activity) was determined using the Alamar blue assay. EGTA (2.5 mM) was also included (with/without serum). The results shown are mean values ± S.D. Data were analyzed using two-way ANOVA followed by Tukey's *post hoc* analysis. *$p<0.05$, ****$p<0.0001$. For TEER measurements under the same conditions, see Figure 6.
